# Nervous System-on-Chip: Innovative Microfluidic Platform to Compartmentalize hiPSC-Derived Neural Networks

**DOI:** 10.3390/mi17020199

**Published:** 2026-02-01

**Authors:** Rahman Sabahi-Kaviani, Antigoni Gogolou, Celine Souilhol, Mark van der Kroeg, Steven A. Kushner, Femke M. S. de Vrij, Anestis Tsakiridis, Regina Luttge

**Affiliations:** 1 Neuro-Nanoscale Engineering, Department of Mechanical Engineering/Microsystems and Institute of Complex Molecular Systems, Eindhoven University of Technology, 5600 MB Eindhoven, The Netherlands; r.sabahi.kaviani@tue.nl; 2 Centre for Stem Cell Biology, School of Biosciences, The University of Sheffield, Western Bank, Sheffield S10 2TN, UKa.tsakiridis@sheffield.ac.uk (A.T.); 3 Neuroscience Institute, The University of Sheffield, Western Bank, Sheffield S10 2TN, UK; 4 Department of Psychiatry, Erasmus MC, University Medical Center, 3015 GD Rotterdam, The Netherlands; m.vanderkroeg@erasmusmc.nl (M.v.d.K.); s.kushner@erasmusmc.nl (S.A.K.); f.devrij@erasmusmc.nl (F.M.S.d.V.); 5 Department of Psychiatry, Columbia University Irving Medical Center, New York, NY 10032, USA; 6 ENCORE Expertise Center for Neurodevelopmental Disorders, Erasmus MC, University Medical Center, 3015 GD Rotterdam, The Netherlands; 7 Eindhoven Artificial Intelligence Systems Institute and Casimir Institute, Eindhoven University of Technology, 5600 MB Eindhoven, The Netherlands

**Keywords:** nervous system-on-chips (NoCs), microtunnel devices (MDs), cortical neural networks, enteric neural networks, biomaterials, nanogrooves, differentiation, human induced pluripotent stem cells (hiPSCs)

## Abstract

This study presents the development of a Nervous System-on-Chip (NoC) using microfabrication techniques, focusing on the integration of human induced pluripotent stem cell (hiPSC)-derived neurons. We designed and fabricated NoCs based on microtunnel devices (MDs) with radial and linear configurations to facilitate the compartmentalized culture of cortical and enteric neural networks. Our findings demonstrate that these MDs allow axonal growth while restricting migration of somas and dendrites between compartments, thereby promoting the formation of organized neural networks. This creates a microfluidic platform capable of supporting the growth of different culture systems, which could potentially be combined to study interactions between the central and enteric nervous systems. The resulting neuronal networks exhibited viability, expression of key lineage markers, and synapse formation, highlighting the platform’s potential for advanced nervous system modeling. MD-based NoC models provide an innovative microfluidic platform for studying the biology of human neural networks, with implications for the investigation of neurodegenerative diseases such as Parkinson’s Disease and applications in pre-clinical research.

## 1. Introduction

The complexity of the human nervous system, encompassing both the central nervous system (CNS) and the peripheral nervous system (PNS), presents significant challenges to researchers, particularly in understanding the communication and interaction between these interconnected components. Modeling the link between the CNS and PNS is crucial for advancing our knowledge on neurological disease pathology, drug discovery, and toxicity [1,2]. Investigating such connectivity is especially important in cases where retrograde modulations or disruptions within the nervous system contribute to conditions such as neurodegenerative disorders (NDDs) [3]. However, current nervous system models are limited in their ability to fully replicate these connections, hindering the effective study of NDDs and other neurological conditions.

Recently, micro- and nanofabrication techniques have enabled the development of a Nervous Systems-on-Chip (NoC), which provides an in vitro representation of the human nervous system and mimics critical aspects of its microenvironment [4,5,6,7]. NoCs incorporate multiple cell types within microfluidic platforms, enabling the formation of complex neural networks [8,9]. These models benefit from the controlled microenvironments that are vital for studying neural connectivity and function. A key approach to creating such environments involves the use of microfluidic compartmentalization [10,11,12], specifically with microtunnel devices (MDs) [13]. MDs consist of small, carefully designed tunnels that guide neurite outgrowth between reservoirs containing spatially confined neuronal networks. These devices are beneficial for studying the interactions between different types of neurons or between neurons and other cell types. They could further facilitate studies of disease initiation and progression, for example, to better understand Parkinson’s Disease [14]. Microtunnels are already used in neuroscience studies across various domains, including axotomy and neuronal injury studies [15,16,17], electrophysiological recording [18,19,20,21], and neurodegenerative disease models [22]. Previously, microtunnels have been employed to investigate retrograde transport and electrophysiological responses relevant to the gut–brain axis using rat vagal afferent neurons [23]. However, for a more physiologically relevant representation of connectivity, it is essential to incorporate human cells into these models.

In this study, we build upon our earlier research [13] by integrating human induced pluripotent stem cell (hiPSC)-derived neurons into an NoC. While similar systems have been reported in the literature [24,25], our focus is on exploring the culture performance of different neuronal cell types, in particular, frontal cortical and enteric neurons, by utilizing MD-based NoCs [13] to guide their organization into networks. Our testing approach further details insights into the influence of sealing substrate materials and topography on enteric neuron differentiation.

In addition to the existing tunnel-based platforms, we specifically chose human iPSC-derived neurons using the reference WTC-11. They form mature, synaptically connected human networks whose morphology and electrophysiology are substantially close to primary neurons. In contrast, commonly used neuronal cell lines exhibit limited excitability, atypical network organization, and restricted capacity to model human genetic backgrounds, limitations that would likely underestimate the functional capabilities and intended use-cases of an NoC. The WTC-11 line provides a well-characterized and ethically sound approach compared to embryonic stem cell sources. These advantages make cells derived from this source particularly suitable for their integration in human disease modeling technology.

Collectively, our data suggest that this innovative microfluidic platform to compartmentalize hiPSC-derived neural networks may lead to a more complete model of the human nervous system.

## 2. Materials and Methods

### 2.1. Cell Culture and Differentiation

All experiments were carried out using the WTC-11 human iPSC line [provided by Bruce R. Conklin (The Gladstone Institutes and UCSF), RRID:CVCL-Y803] [26,27]. Cells were routinely screened for expression of pluripotency markers, mycoplasma testing and karyotypic abnormalities using either the Infinium Global Screening Arrays (Illumina, San Diego, CA, USA) or digital PCR [28].

Prior to the generation of cortical neural progenitor cells (NPCs), the hiPSCs were cultured on a feeder layer of mouse embryonic fibroblasts according to Gunhanlar et al. [28]. Prior to enteric neuron differentiation, hiPSC cultures were routinely performed in feeder-free conditions in mTeSR1 medium (Stem Cell Technologies) on Geltrex LDEV—free reduced growth factor basement membrane matrix (Thermo Fisher, Waltham, MA, USA, #A1413202). Cells were passaged twice a week after reaching approximately 80% confluency using PBS/EDTA (Invitrogen, Carlsbad, CA, USA) or ReleSR (Stem Cell Technologies) as passaging reagents. hiPSCs and their differentiated derivatives were maintained at 37 °C under a humidified atmosphere and 5% CO_2_ levels.

#### 2.1.1. Cortical Neural Network Cultures

To generate cortical neural networks comprising neurons and astrocytes, NPCs were differentiated from WTC-11 hiPSCs using an embryoid body-based protocol [28], with the addition of fluorescence-activated cell sorting (FACS) to enhance the purity of cortical NPCs [29]. Briefly, embryoid bodies were generated from the hiPSCs and were plated down after 1 week. Resulting pre-NPCs were detached from the culture plate using Accutase™ (Stem Cell Technologies, Vancouver, BC, Canada, 07920) and resuspended in a single cell solution after three passages. Resuspended pre-NPCs were purified by FACS to collect CD184+/CD44-/CD271-/CD24+ cells using a FACSaria III (BD bioscience). The purified NPCs were differentiated into neural networks in the MDs according to Gunhanlar et al. [28]. MDs were coated with 100 µg/mL ready-to-use poly-L-ornithine solution (P4957-50 mL, Sigma-Aldrich, St. Louis, MO, USA) for 1 h at room temperature. The wells were washed 3 times with dH_2_O and allowed to air dry. The poly-L-ornithine-coated wells were subsequently coated with laminin (50 µg/mL in dH_2_0) for 30 min at 37 °C. The NPCs were dissociated with Accutase (Stem Cell Technologies) and seeded in the MDs in Neural Differentiation Medium according to Gunhanlar et al. [28] with 1500 NPCs per 3 mm-diameter well and 750 NPCs per 2 mm-diameter well. Neural Differentiation Medium: Neurobasal medium (Thermo Fisher Scientific), 1% N2 supplement (Thermo Fisher Scientific), 2% B27-RA supplement (Thermo Fisher Scientific), 1% minimum essential medium/non-essential amino acid (Stem Cell Technologies), 20 ng/mL brain-derived neurotrophic factor (ProSpec Bio, Rehovot, Israel), 20 ng/mL glial cell-derived neurotrophic factor (ProSpec Bio), 1 μM dibutyryl cyclic adenosine monophosphate (Sigma-Aldrich), 200 μM ascorbic acid (Sigma-Aldrich), 2 μg/mL laminin (Sigma-Aldrich) and 1% penicillin/streptomycin (Thermo Fisher Scientific). Cells were refreshed every 2–3 days.

#### 2.1.2. Generation of Enteric Nervous System Cells

Generation of enteric neurons from iPSCs involved three successive steps as previously described [30,31]: (1) Generation of vagal neural crests (NC)/early enteric nervous system (ENS) progenitors; (2) further derivation/maturation of ENS progenitors as spheres; (3) specification of enteric neurons and glia in 3D or 2D cultures on chips or coverslips.

For vagal neural crest differentiation, WTC-11 iPSCs were dissociated using Accutase (Merck Life Science, Darmstadt, Germany, #A6964) and replated at a density of 30,000 cells/cm^2^ on Geltrex-coated 6-well culture plates directly into an NC inducing medium consisting of DMEM/F12 (Merck Life Science, #D6421), 1 × N2 supplement (Thermo Fisher Scientific, #17502048), 1 × GlutaMAX (Thermo Fisher Scientific, #35050038), 1 × MEM NEAA(Thermo Fisher Scientific, #11140050), the TGF-beta/Activin/Nodal inhibitor SB-431542 (2 μM, Tocris, Bristol, UK, #1614), CHIR99021 (1 μM, Tocris, #4423), BMP4 (20 ng/mL, Thermo Fisher Scientific, #PHC9534), the BMP type-I receptor inhibitor DMH-1 (1 μM, Tocris, #4126), and ROCK inhibitor Y-27632 2HCl (10 μM, AdooQ Bioscience, Irvine, CA, USA, #A11001), with the latter being withdrawn from the differentiation medium after the second day of NC induction. The day of plating is considered day 0 of differentiation. At day 4 of differentiation, the medium was changed and supplemented with 1 μM all-trans retinoic acid (RA) (Merck Life Science, #R2625), followed by replenishment with freshly RA-supplemented medium on day 5.

Day 6 vagal NCs were replated in non-adherent conditions at a 1:1 ratio to form free-floating spheres using the Corning Ultra-Low attachment plates (Corning, Corning, NY, USA, #3471). Spheres were cultured for 3 days in 0.5 × DMEM/F12, 0.5 × Neurobasal medium (Thermo Fisher Scientific, #21103049), 1 × N2 supplement, 1 × B27 supplement (Thermo Fisher Scientific, #17504001), 1 × GlutaMAX, 1 × MEM NEAA, CHIR99021 (3 μM), FGF2 (10 ng/mL, R&D systems, #233-FB/CF), all-trans RA (1 μM) and ROCK inhibitor Y-27632 2HCl (10 μM). At day 8, 48 h post plating, spheres of different sizes were formed, and sphere media was half changed, carefully, using a serological pipette to avoid excessive cell loss.

Day 9 spheres were either transferred in Matrigel on the chips (3D cultures) and cultured for an extra 30 days, plated on Geltrex-coated culture plates (2D cultures) or plated on Geltrex-coated modified coverslips (1:3 or 1:4 ratio) and cultured for an extra 11 days in ENS induction medium. After 26 days in culture (day 35 neurons), the 2D cultures were replated on the chips and cultured for at least 30 more days. For replating, neurons were washed twice with PBS and incubated with Accutase for 20 min at 37 °C. DMEM was added, cells were gently dissociated by pipetting to preserve network integrity and then transferred to a 50 mL Falcon tube. Neurons were allowed to settle by gravity for ~3 min (no centrifugation), and excess medium was carefully removed. The pellet was gently resuspended in ENS induction medium and plated onto Geltrex-coated MDs.

For differentiation on coverslips, all coverslips were first sterilized with 70% ethanol, then thoroughly washed with PBS. They were placed into empty 12-well plates and incubated for a couple of hours or overnight at 37 °C to dry. Then, Geltrex was added to each well in a volume sufficient to fully cover the coverslip and the surrounding well surface. Plates were returned to the incubator and left overnight at 37 °C. The ENS induction medium contains Brainphys (Stem Cell Technologies, #05790), 1 × N2 supplement, 1 × B27 supplement, 1 × GlutaMAX, 1 × MEM NEAA, GDNF (10 ng/mL, Peprotech, #450-10), DAPT (10 μM, Tocris, #2634) and L-Ascorbic acid 2-phosphate sesquimagnesium salt hydrate (200 μM, Sigma-Aldrich, #A8960) and was changed every other day. Prior to culture in chips, the devices were rinsed with 70% ethanol and then coated first with poly-L-ornithine (P4957-50 mL, Sigma-Aldrich) and then Geltrex.

### 2.2. Immunostaining and Imaging

For the live staining of hiPSC-derived cortical neural network cultures, neural cultures were incubated with the LIVE dye of the LIVE/DEAD™ Viability/Cytotoxicity Kit according to the manufacturer’s instructions (Thermo Fisher Scientific). For immunostaining, the neural cultures in the MDs were fixed for 20–30 min using 4% formaldehyde in phosphate-buffered saline (PBS), washed with PBS 3× 10 min, and blocked for 1 h by pre-incubation in staining buffer containing 0.05 M Tris, 0.9% NaCl, 0.25% gelatin and 0.5% Triton-X-100 (pH 7.4). Primary antibodies were incubated for 48–72 h at 4 °C in staining buffer, washed with PBS, followed by incubation with the secondary antibodies in staining buffer for 2 h at room temperature. The cultures were embedded in Mowiol 4-88 (Sigma-Aldrich), after which confocal imaging was performed with a Zeiss LSM800 confocal microscope using ZEN software, Version 2.6 (Zeiss, Oberkochen, Germany). The following primary antibodies were used: Synapsin 1/2 (Synaptic Systems 106004, Göttingen, Germany, 1:200); NF200 (Sigma-Aldrich 083M4833 1:200); MAP2 (Synaptic Systems 188004, 1:200); Tau (Cell Signaling Technology 4019, Danvers, MA, USA, 1:200); and DAPI (Thermo Fisher Scientific D1306). The following secondary antibodies were used 1:200: Alexa-488, Alexa-555, and Alexa-647 (Jackson Immuno Research, West Grove, PA, USA).

ENS cells were fixed in 4% paraformaldehyde (PFA) for 10 min at room temperature, rinsed twice with PBS and permeabilized with 0.5% Triton X-100 in PBS containing 10% fetal calf serum (FCS) and 0.1% bovine serum albumin (BSA) for 15 min. Blocking was then performed in a blocking buffer consisting of 10% FCS and 0.1% BSA in PBS at room temperature for 1–2 h or overnight at 4 °C. Primary antibodies were diluted in the blocking buffer, and cells were incubated with primary antibodies overnight at 4 °C. Following three washes, cells were incubated with secondary antibodies conjugated to Alexa fluorophores (Invitrogen) diluted in blocking buffer for 1–2 h at room temperature and in the dark. Cell nuclei were counterstained with Hoechst 33342 (Thermo Fisher Scientific, #H3570, 1:5000). Stained cells grown on coverslips were mounted on 75 mm × 25 mm glass slides (VWR, Menzel Gläser, SuperFrost^®^ Plus) using 8–12 μL of VECTASHIELD mounting media (2B Scientific, #H-1700-10) and left to dry in the dark before imaging or before storing them at 4 °C. The following primary antibodies were used: anti-beta III tubulin (Abcam, #ab78078, 1:1000); anti-S100 (Millipore, #S2532, 1:1000); anti-neurofilament (Abcam, #ab8135, 1:1000); Anti-Alpha-Smooth Muscle Actin (Invitrogen, #14-9760-82, 1:500). Fluorescent images were acquired using the InCell Analyser 2200 system (GE Healthcare, Chicago, IL, USA).

### 2.3. Quantitative Real-Time PCR

To avoid contamination from cells not grown directly on coverslips, residual cells located in the space between the coverslip and the plastic surface of the well were first dislodged by gentle scraping with a P200 pipette tip and thoroughly washed off with PBS. Following this step, cells cultured on the modified coverslips were detached using Accutase and collected for RNA extraction. Total RNA was extracted using the total RNA purification kit (Norgen Biotek) following the manufacturer’s instructions. CDNA synthesis was performed using the High-Capacity cDNA Reverse Transcription Kit (Thermo Fisher). Quantitative real-time PCR was carried out using the QuantStudio 12 K Flex (Applied Biosystems, Foster City, CA, USA) thermocycler in combination with the Roche Universal Probe Library system and the TaqMan™ Fast Universal PCR Master Mix (Applied Biosystems) following manufacturer instructions. Primer sequences and corresponding probes are shown in Table S1. Graphs were generated using GraphPad Prism (GraphPad Software, Version 10), which was also employed for statistical analysis.

### 2.4. Device Preparation

In these experiments, we utilized MDs to establish a compartmentalized environment for studying cortical and ENS cultures. Additionally, to explore the effects of different materials and topographical features on the differentiation of the stem cell-derived neuronal networks, we fabricated and tested flat and nanogrooved substrates. These substrates were made from polydimethylsiloxane (PDMS) and Norland Optical Adhesive 81 (NOA81, Norland Products Inc., Jamesburg, NJ, USA). These materials have previously been employed in the microfabrication of Brain-on-Chip components [32,33,34,35,36].

#### 2.4.1. Nanogrooves

PDMS nanogrooves were prepared by nanoimprint lithography according to specifications first described by Xie and Luttge [37]. Periodicity (D) ranged from 200 to 2000 nm and ridge widths (L) from 100 to 1340 nm in the original quartz stamp, which was kindly provided by the Bijkerk group at the University of Twente, The Netherlands. Upon extending the mold fabrication process [38] to a more durable secondary mold, a cyclic olefin copolymer (COC) master was obtained.

The PDMS elastomer and cross-linking agents (SYLGARD 184, Dow Corning, Midland, MI, USA) were mixed at a ratio of 10:1 and degassed for 20 min in a vacuum desiccator. PDMS mixture was then poured and spin-coated on the COC master mold at 500 rpm for 30 s with acceleration of 200 rpm/s and cured in a 65 °C oven for 4 h prior to peeling it from the COC template. The nanogrooves were then either used as substrates to be bonded to MDs or as a PDMS working mold in the microtransfer molding of NOA81 nanogrooves on coverslips [39].

#### 2.4.2. MD Design

**Figure 1:**
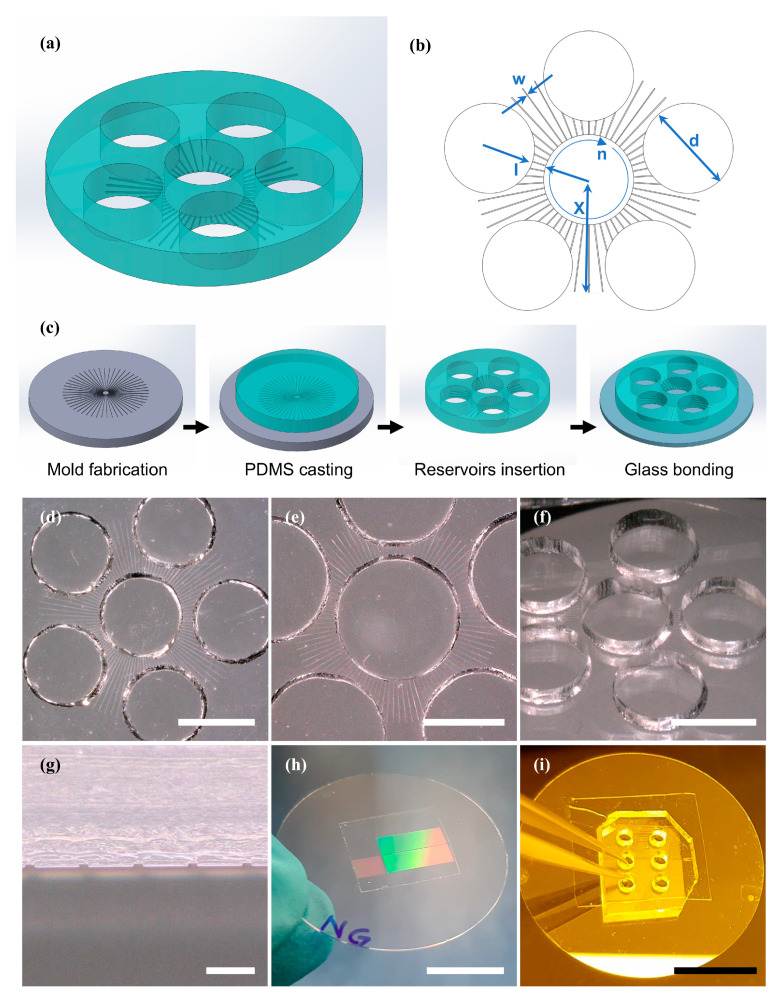
Design and fabrication of MD. (**a**) 3D schematic of the radial MD design. (**b**) Top view of the radial MD design, highlighting the key design parameters: reservoir diameter (d), compartment distance (l), maximum tunnel length from the center (X), tunnel width (w), and the total number of tunnels (n). (**c**) The fabrication steps for the MD made of PDMS using soft lithography. (**d**,**e**) Top-view photographs of the fabricated radial MD on a glass microscope coverslip, with 2 mm and 3 mm reservoirs, respectively (scale bars: 2 mm). (**f**) Photograph of a radial MD with 2 mm reservoirs from an angle (scale bar: 2 mm). (**g**) Cross-sectional view of tunnels in a PDMS MD showing the openings through which axons pass (scale bar: 50 µm). (**h**) Photograph of a PDMS nanogroove on a coverslip, where groove patterns are visible through light interference colors (scale bar: 10 µm). (**i**) Photograph of a linear MD bonded to a flat PDMS layer, attached to a glass coverslip (scale bar: 10 mm).

Figure 1a presents a 3D-schematic drawing of the radial MD design and Figure 1b shows a top view detailing the design parameters. The reservoir diameter (d) was set at 2 mm or 3 mm. The tunnel width (w) was limited to 10 µm, a constraint determined by resolution capabilities yet narrow enough to prevent large axonal bundles from entering the tunnel. The tunnel height, corresponding to the SU-8 mold thickness, was designed to be 6 µm and 4 µm. The reduced thickness was fabricated to prevent cell somas from slipping through the tunnels and migrating to the other compartment while still maintaining functional integrity.

The furthest point on the tunnels from the center (X) is 2.5 mm, allowing exploration of different ranges of tunnel distance between the central and surrounding reservoirs. The tunnel lengths between reservoirs (minimal compartment distances) were at 300 µm during this study. The total number of tunnels (n) is 100. The tunnels are radially evenly spread around the central compartment. There are five additional compartments arranged such that they surround the central compartment, each at an equal distance.

The linear MD design consisted of parallel tunnels with a total length of 25 mm and tunnel widths of 10, 15, and 20 µm, with tunnel pitches ranging from 60 µm to 120 µm. The layout of the photomask for both the radial and linear MD designs can be found in Supplementary Materials Figure S1.

#### 2.4.3. MD Fabrication

The two variations of MDs (linear and radial) were prepared based on the fabrication details found in Bastiaens et al. [13]. The main steps in the fabrication process are depicted in Figure 1c. The full details of the SU-8 2010 (Microchem Inc., Newton, MA, USA) photolithography on a silicon (Si) wafer, which yielded molds for soft lithography [40], can be found in the Supplementary Materials S2. The thicknesses of the photoresist used to make MDs were aimed at a height of 4 µm and 6 µm, respectively. In brief, PDMS was prepared and degassed according to the protocol described in Bastiaens et al. [13]. Subsequently, aluminum foil was wrapped around the 4 in-diameter Si wafer to create a cavity for pouring a defined amount of PDMS onto the mold. We used a 7 g PDMS mixture to gain a PDMS thickness of 1 mm. The PDMS was cured at 65 °C (DL 53 oven, VWR^®^) for 4 h. The PDMS was peeled, and holes for the cell culture reservoirs were punched using a biopsy tool. The Computer Aided Design (CAD) drawings (Figure S2) for the stencil and further details on this step in fabrication are given in the Supplementary Materials S3.

For the cortical neural network cultures, the radial MDs were affixed to oven-pre-heated (8 h at 180 °C, Heraeus T 6 P, Kendro Laboratory Products) 18 mm-diameter glass coverslips. Linear MDs were either affixed directly to a 30 mm-diameter glass coverslip or onto a flat PDMS layer. Knife-cut 15 mm × 15 mm squares of either flat PDMS layer or nanogrooves were first placed onto a 30 mm-diameter glass coverslip (Thermo Scientific, Menzel-Gläser, Germany) and then bonded to MDs for the final assembly of the devices. From the dimensional variations of nanogrooves [37], the pattern marked up with D600L430 was overlaid with the reservoirs of the linear MD pattern for ENS cultures. Prior to completing MDs by direct PDMS bonding [13], the surfaces were activated by oxygen plasma using an EMITECH K1050X plasma asher (Quorum, Laughton, UK) at 20 W for 30 s. The fabricated radial and linear MDs bonded on glass coverslip are shown in Figure 1d–g. Since PDMS is known to alter over time, all biological experiments were started within a few weeks after fabrication.

#### 2.4.4. Large Area Culture Substrates for qPCR

To explore the influence of substrate material and topography on stem cell differentiation, additional flat and nanogrooved substrates were fabricated from PDMS and NOA81, offering a larger culture area compared to the actual MDs. These substrates facilitate the collection of sufficient cell material for gene quantification.

The flat PDMS substrates were created by spincoating the prepared and degassed PDMS, according to the protocol described above, onto a 4-inch silicon wafer at 500 rpm for 30 s. The PDMS was then cured, cut, and placed on an 18-mm glass coverslip (VWR^®^, Darmstadt, Germany). The nanogrooved PDMS substrate was fabricated using replica molding on a COC mold as previously described in Section 2.4.1, and then cut and mounted on an 18-mm coverslip. For this set of devices, a COC mold fabricated using laser interference lithography [41] was utilized. The nanogrooves on this substrate were measured to have a pattern periodicity of 600 nm and a ridge width of 330 nm (D600L330).

The method of fabrication can influence the material properties; therefore, NOA81 substrates were fabricated using three different techniques. The first set of a simply flat substrate was fabricated by spin-coating NOA81 directly onto 18-mm glass coverslips at 2000 rpm for 30 s. This was followed by curing under UV light using the UV-LED exposure system (IDONUS, UV-EXP 150R, Neuchatel, Switzerland) with an energy dosage of 8000 mJ/cm^2^ at an intensity of 20 mW/cm^2^. The other set of a flat substrate was fabricated using microtransfer molding (μTM), a technique that also enables the creation of surface features in NOA 81 films or foils [39]. To also include the potential effects of processing choice on surface quality, a flat PDMS substrate was used as a working mold. It was made hydrophilic through oxygen plasma treatment in a plasma asher at 3 W for 30 s. NOA81 was drop-cast onto the PDMS mold, spin-coated at 600 rpm for 30 s, and then transferred onto an 18-mm glass coverslip. The assembly received an initial UV energy dosage of 4000 mJ/cm^2^ at an intensity of 20 mW/cm^2^. After peeling off the PDMS mold, the NOA81 substrate received an additional 4000 mJ/cm^2^ energy dosage.

Accordingly, the nanogrooved NOA81 substrates were fabricated using μTM. A nanogrooved PDMS working mold was created from a COC mold as described above, by spin-coating PDMS at 500 rpm for 30 s. The PDMS mold was then rendered hydrophilic using a plasma asher with the same parameters as above, followed by drop-casting with NOA81. The NOA81 was spin-coated at 1000 rpm for 30 s, a slightly higher speed than used for the flat mold, to ensure complete coverage of the nanogrooves. Like for the flat NOA81 films, the assembly was transferred to an 18-mm glass coverslip and subjected to an initial UV energy dosage of 4000 mJ/cm^2^, followed by peeling off the PDMS mold and an additional 4000 mJ/cm^2^ energy dosage.

To ensure complete curing and eliminate any potential toxicity to cell cultures, all NOA81 substrates underwent an additional UV energy dosage of 10,000 mJ/cm^2^ at an intensity of 20 mW/cm^2^.

## 3. Results and Discussion

### 3.1. Generation of Neural Networks in Microtunnel Devices

We aimed to investigate if neural networks derived from a hiPSC line (WTC-11) [26,27] are biocompatible with a microtunnel device (MD) previously tested for use with the SH-SY5Y cell line [13]. We elaborated on their construction materials and various NoC design parameters, like the number and arrangement of reservoirs, as well as different sealing materials and topographies.

#### 3.1.1. Cortical Neural Networks

**Figure 2:**
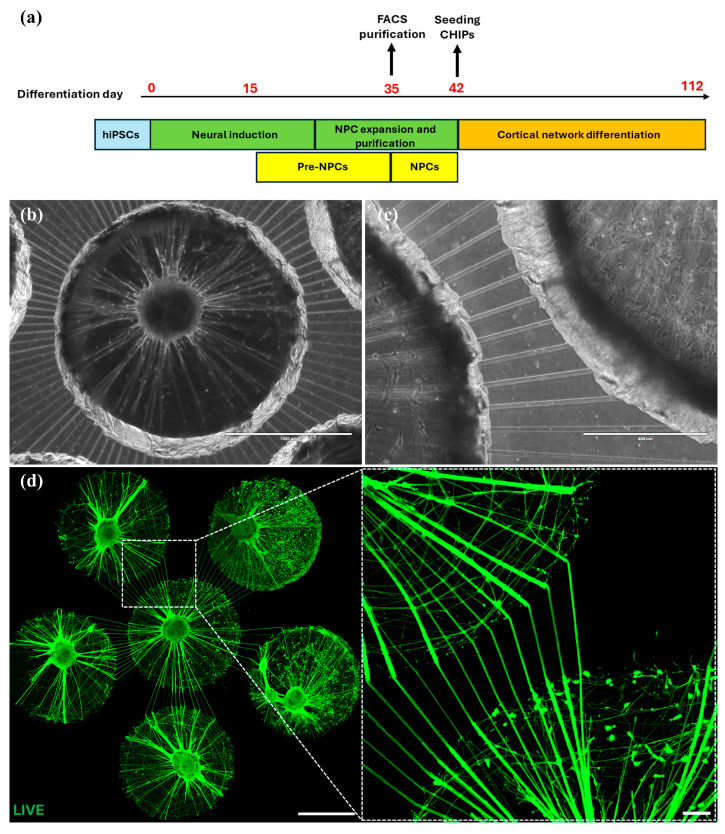
Human iPSC-derived neurons in the microtunnel device. (**a**) Schematic depiction of the key stages of our hiPSC differentiation protocol towards cortical neural networks and the timing of their integration within the chip devices (**b**) Neural network culture day 50 in 2 mm wells (scale bar: 1 mm), (**c**) neurons projecting into neighboring wells that do not contain any cells, only medium (scale bar: 400 µm), (**d**) neural network culture day 50 in 2 mm wells with LIVE dye in green. Neurons project to neighboring neural cultures through the microtunnels (scale bars: left, 1 mm; right, 100 µm).

**Figure 3:**
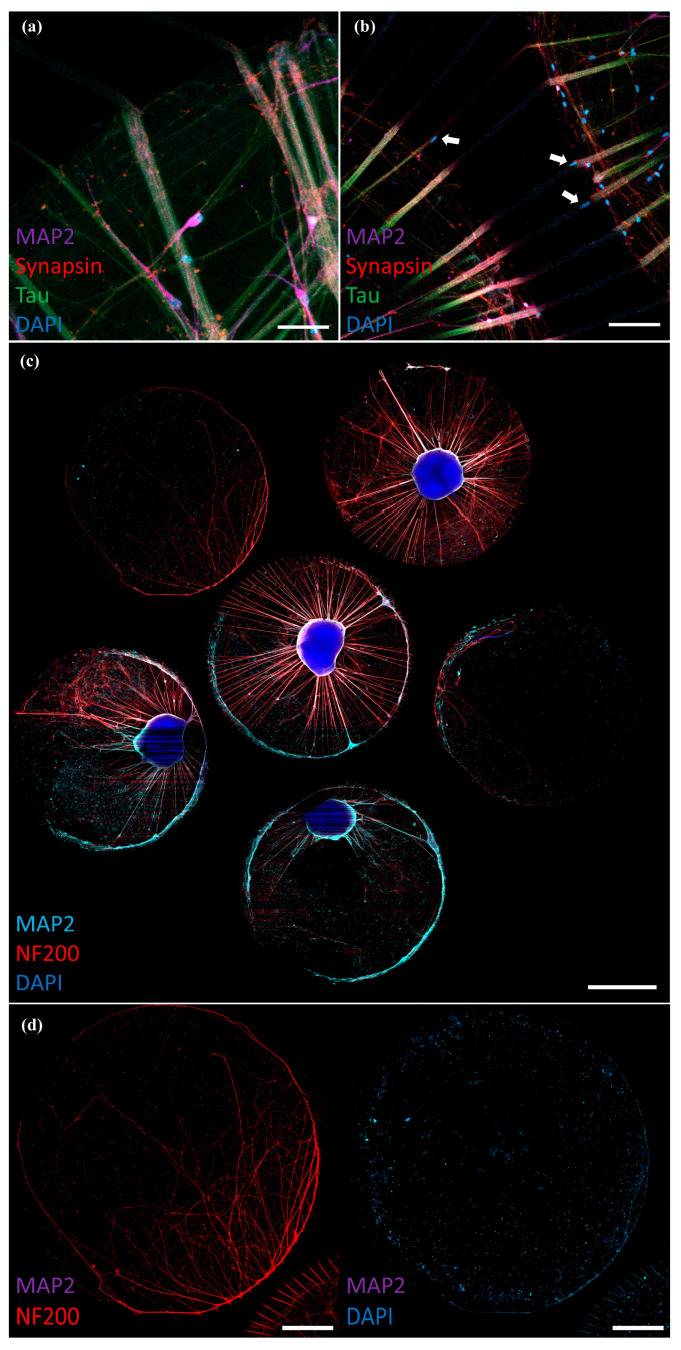
Human iPSC-derived neurons in the microtunnel device. (**a**) Day 70 neural networks in the microtunnel device. The culture contains MAP2+ neuronal dendrites, Tau+/MAP2− axons and synapsin+ pre-synaptic terminals; 3 mm well microtunnel device (scale bar: 50 µm), (**b**) day 70 neural network, white arrows show DAPI+ nuclei at the entrance or partially in the tunnels designed with a width of 10 µm diameter and 6 µm height; 3 mm well microtunnel device (scale bar: 100 µm), and (**c**) overview of a whole 3 mm well microtunnel device with neural cultures on day 70. Some wells were kept empty to determine axonal outgrowth (scale bar: 1 mm). (**d**) Day 70 neural cultures with NF200+ axons growing into empty wells, but no MAP2+ dendrites (emphasized in the right panel of the same field of view showing lack of signal in the MAP2 channel); tunnel width 10 µm, tunnel height 4 µm; 3 mm well microtunnel (scale bars: 500 µm).

To this end, hiPSC-derived cortical neural network cultures (Figure 2a–c) were generated in a unique NoC configuration connecting six reservoirs sealed on glass (Figure 1f) by adapting a published protocol for hiPSC-derived neural networks [28]. The resulting network consisted of neurons and glia obtained from a common frontal cortical patterned neural progenitor pool. Cells were able to extend into the tunnels and project to neighboring wells (Figure 2b–d). The neuronal identity of the cultures was verified by immunostaining, revealing positivity for the neuronal markers MAP2 and Tau (Figure 3a,b). Expression of the presynaptic terminal marker Synapsin further demonstrated the formation of synapses in the culture (Figure 3a,b).

In the initial versions of the MDs, tunnels had a width of 10 µm and a height of approximately 6 µm, as confirmed by a quick profilometer scan of the microstructures on the mold without detailed analysis. In these earlier versions, DAPI+ nuclei were present in the tunnels, indicating that cell somas were able to enter the microtunnels rather than only axons (Figure 3b).

Keeping the tunnel width of 10 µm in the mask design, we optimized the tunnel height to approximately 4 µm (by evaluating spin-coat parameters during SU-8 mold fabrication). These tunnel dimensions were found to be sufficient to prevent cell somas from entering the tunnels. With these tunnel dimensions (confirmed from a profilometer scan of the features on the mold, data shown in Figure S3 in the Supplementary Materials), only axons (NF200+, MAP2−) grew into neighboring wells (Figure 3d). The tunnel length did not have a noticeable impact on axonal extension into the adjacent compartment, as no clear preference was observed between shorter (~300 µm) and longer (~700 µm) tunnels (Figure 2d). We did not yet analyze potential differences in synapse numbers in relation to tunnel length or height in this study; however, it could be possible that for shorter tunnels, there is a tendency that dendrites can also grow through the tunnels, which may affect the synaptic density.

In these MDs, NF200+ axons were found to enter the tunnels and project into the neighboring wells with axon lengths of up to 5 mm (Figure 3d). Figure 3c shows an overview of a whole 3 mm well microtunnel device with neural cultures on day 70. Some wells were kept empty to determine axonal outgrowth. The axons projected both into wells containing other cortical networks and into empty coated wells that only contained Neural Differentiation Medium without cells.

In comparison to prior compartmentalized microfluidic platforms, this work advances the field by introducing an adaptable, multi-compartment microtunnel architecture that supports adherent hiPSC-derived neural network formation. Unlike previously reported systems that are typically limited to dual-chamber layouts with fixed geometries [25], our platform enables rapid and reproducible customization of well number, well diameter, and tunnel arrangement by punching wells of different diameters using a biopsy tool, while maintaining high positional accuracy across repetitive devices through the use of laser-cut stencils during punching. For this proof-of-feasibility study, the chambers were selected to be 2 and 3 mm in diameter in favor of the adherent cortical neuronal network approach previously developed by Erasmus MC in a 384-well plate format [42], operating without the addition of thick layers of animal-derived scaffolding materials. While wells in standard 384-well plates are typically squared with rounded corners, we explored whether circular wells punched in PDMS could equally facilitate the formation of layered adherent cortical organoids in a compartmentalized configuration, as described previously [42].

Compared to the conventional 384-well plate format, the PDMS microtunnel devices mounted on glass coverslips exhibit a distinct neural network organization within each well. On the one hand, this difference in organization may be considered a limitation of the current approach. On the other hand, it offers an opportunity to further optimize such adherent cortical cell culture protocols toward higher complexity, including co-culturing forebrain-like networks with multiple neuronal cell types and even neural networks representing different regions; for example, by mixing with glial cells and/or applying different inhibitors or modulators to adjust neuronal cell ratios.

#### 3.1.2. Enteric Neural Networks

**Figure 4:**
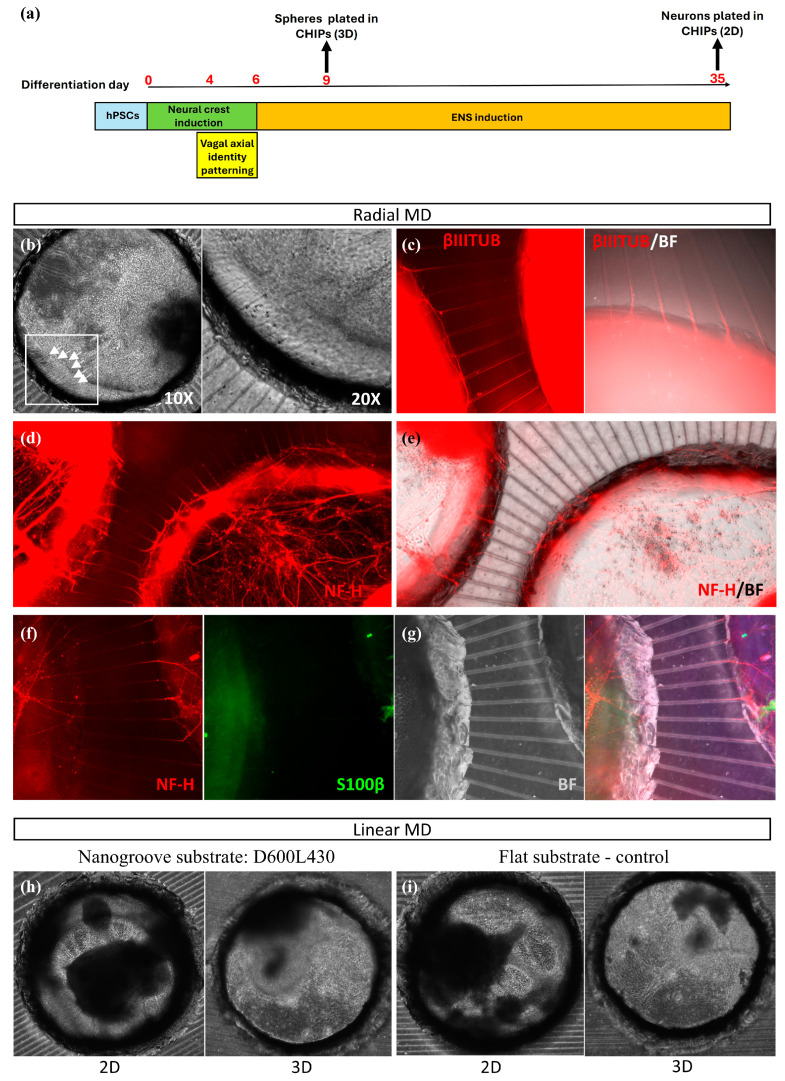
Human iPSC-derived enteric neurons in the microtunnel device. (**a**) Scheme depicting the key stages of our hiPSC differentiation protocol toward ENS-like cells and the timing of their integration within the chip devices. (**b**) Representative brightfield image of a day 9 ENS progenitor sphere plated and cultured for 30 days in the compartment of the radial microtunnel device (microtunnel width = 10 µm), following embedding in Matrigel. The image on the right is a magnified version of the inset in the left image. Arrowheads indicate neuronal processes going through the microtunnels. (**c**) Immunofluorescence images showing βIII-tubulin-positive neuronal processes exiting a compartment containing a day 9 ENS progenitor sphere through the microtunnels of a radial chip device and projecting toward an adjacent sphere-containing compartment. High autofluorescence due to the presence of Matrigel can be observed where the device compartments are located. BF, brightfield. (**d**,**e**) Immunofluorescence images showing neurofilament heavy chain (NF-H)-positive neuronal processes exiting a compartment containing day 35 adherent ENS cultures through the microtunnels of a radial chip device and projecting toward an adjacent sphere-containing compartment after 30 days in culture. (**f**,**g**) Same as (**d,e**) but at higher magnification and including immunofluorescence expression analysis of the glial marker S100β. (**h**,**i**) Bright field images of ENS cells cultured either on a thin Matrigel layer (2D) or embedded in Matrigel (3D) on a linear microtunnel device on nanogrooved PDMS (D600L430) (**h**) and on flat PDMS as a control (**i**).

We next tested the compatibility of our PDMS-based MD designs and sealing substrate materials with PNS neurons, specifically enteric neurons. WTC-11 hiPSCs were differentiated toward enteric neurons and glia using our established protocol [30,31] (Figure 4a) and then re-plated and cultured within various NoC configurations.

First, we examined the ability of both day 9 three-dimensional (3D) ENS progenitor spheres as well as later (day 35 of differentiation) adherent two-dimensional (2D) ENS cultures (Figure 4a) to survive, differentiate (in the case of progenitors) and establish neuronal projections following re-plating in two distinct MD designs: (i) the radial design with six connected reservoirs as for the cortical cell cultures with microtunnels of a width of 10 μm (Figure 1f) and (ii) the linear design (Figure 1g) on flat and nanogrooved PDMS sealing substrates. The radial design was intended to maximize neurite convergence from the central well toward multiple compartments, which is beneficial for organoid-like phenotypes, while the linear design aimed to guide directional neurite outgrowth for more controlled, measurable projections.

Using the various chips with a designed tunnel height of 6 μm, cells were either plated as adherent cultures on a thin layer of Matrigel (day 35 ENS cells) or embedded in Matrigel to grow as 3D aggregates (day 9 ENS progenitor spheres) for up to 30 days (Figure 4a). We found that both Matrigel-embedded βIII-tubulin (TUJ1)+ neuron-projecting spheres (Figure 4b,c) and NF-H-positive adherent neurons (Figure 4d–g) survived well in all devices tested, establishing extended neuronal projections into the microtunnels. Moreover, we detected the presence of S100β+ enteric glial cells (Figure 4f,g).

Similar to what has been observed for cortical neuronal networks, we did not observe a visible influence of tunnel length on ENS axonal extension or differentiation. Specifically, no clear differences were observed in axonal extension into neighboring compartments when comparing shorter (~300 µm) and longer (~700 µm) tunnels.

We also confirmed the biocompatibility of our ENS cell cultures with linear MDs on flat PDMS (Figure 4i) as well as nanogrooved PDMS (Figure 4h). In these figures, the MDs used for 2D culture featured tunnels with a width of 10 µm and a pitch of 60 µm, while those used for 3D culture had a width of 20 µm and a pitch of 70 µm. While both 3D progenitor spheres and 2D adherent cultures survived up to 30 days without replating and established extended neuronal networks in all tested MD configurations, the effect of these MD designs on the lineage identity of the cells was not characterized. A more detailed analysis of potential differences in gene expression related to the substrate materials and topographies is addressed in the next section (Section 3.2).

The microtunnel device provides a versatile platform to study axonal development, signaling, and connectivity under controlled conditions. It allows co-culture of distinct PSC-derived neuronal or non-neuronal populations that communicate only through axons, eliminating direct somatic contact. This setup is especially valuable for modeling long-range circuit interactions between CNS or ENS regions, and for combining control and disease-derived cells to study how genetic backgrounds or pathological conditions influence axon-mediated communication and synapse formation. Additionally, specific disease models can be envisaged, where specific organoids are innervated, such as, for example, in the case of ENS-innervated co-cultured gut organoid models [43,44]. Moreover, the platform can be deployed to examine crosstalk between tumoroids and various types of innervation from the PNS.

The architecture of the formed neural network derived from the WTC-11 cell line introduces several limitations, particularly when compared with 3D organoid models. In contrast to 3D organoids or assembloids, which can self-organize into complex cytoarchitectures and support long-range interactions across multiple axes, the MD setup purposefully imposes structural constraints to limit cell contact and migration between compartments. As a result, the system primarily focuses on axon projections from one defined cell source or tissue (for instance, ENS cells or a specific brain region such as the thalamus or cortex) toward another. While the MD is a less complex model compared to a fully 3D whole organoid system, it benefits from reduced variability and higher reproducibility.

The platform offers exciting future developments, such as its extension for Parkinson’s Disease (PD) modeling by connecting the ENS to midbrain organoids, enhancing our understanding of CNS–ENS interactions. Additionally, the use of adherent cortical networks could provide insights into neurodevelopmental diseases. Future techniques, including neuronal signal sensing via multielectrode arrays (MEAs) or optogenetics, could enable real-time monitoring of neuronal activity, advanced studies of neuronal network functionality and disease mechanisms. Moreover, integrating 3D bioprinting or organoid co-culture systems could create more complex multi-tissue models for studying brain disorders and drug testing.

### 3.2. Assessing the Effect of Substrate Material/Topography Designs on ENS Differentiation

**Figure 5:**
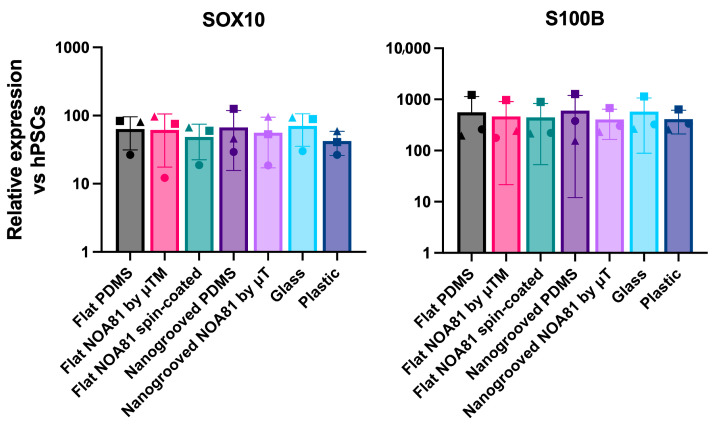
RT-qPCR analysis of expression of ENS progenitor/glial markers in day 21 cultures generated following plating of day 9 spheres on the indicated substrates. Data are presented as mean ± S.D. of three independent experiments. Different shapes represent individual experiments.

**Figure 6:**
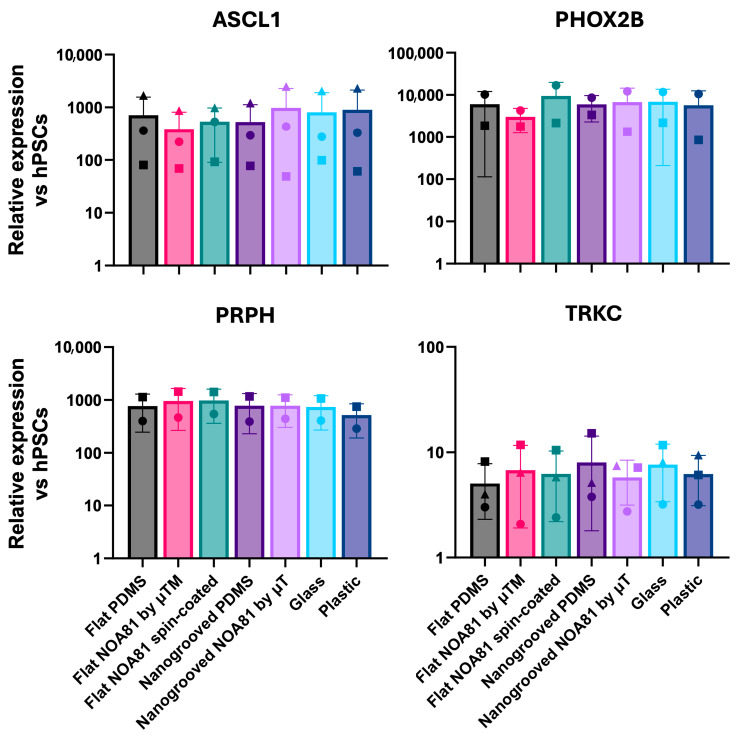
RT-qPCR analysis of expression of enteric neuron markers in day 21 cultures generated following plating of day 9 spheres on the indicated substrates. Data are presented as mean ± S.D. of three independent experiments. Different shapes represent individual experiments. For PHOX2B and PRPH, two experiments were analyzed due to technical constraints, including limited probe availability.

**Figure 7:**
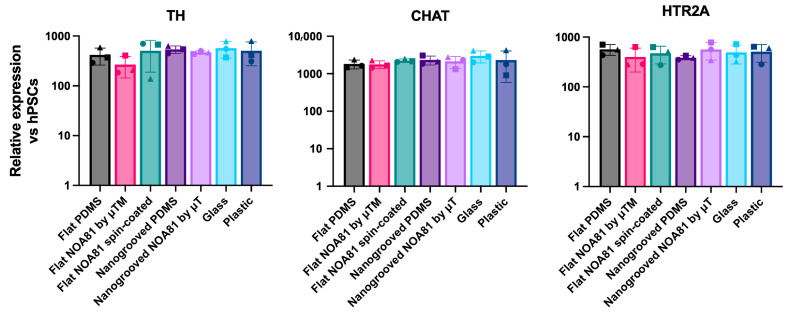
RT-qPCR analysis of expression of enteric neuronal subtype markers in day 21 cultures generated following plating of day 9 spheres on the indicated substrates. Data are presented as mean ± S.D. of three independent experiments. Different shapes represent individual experiments.

**Figure 8:**
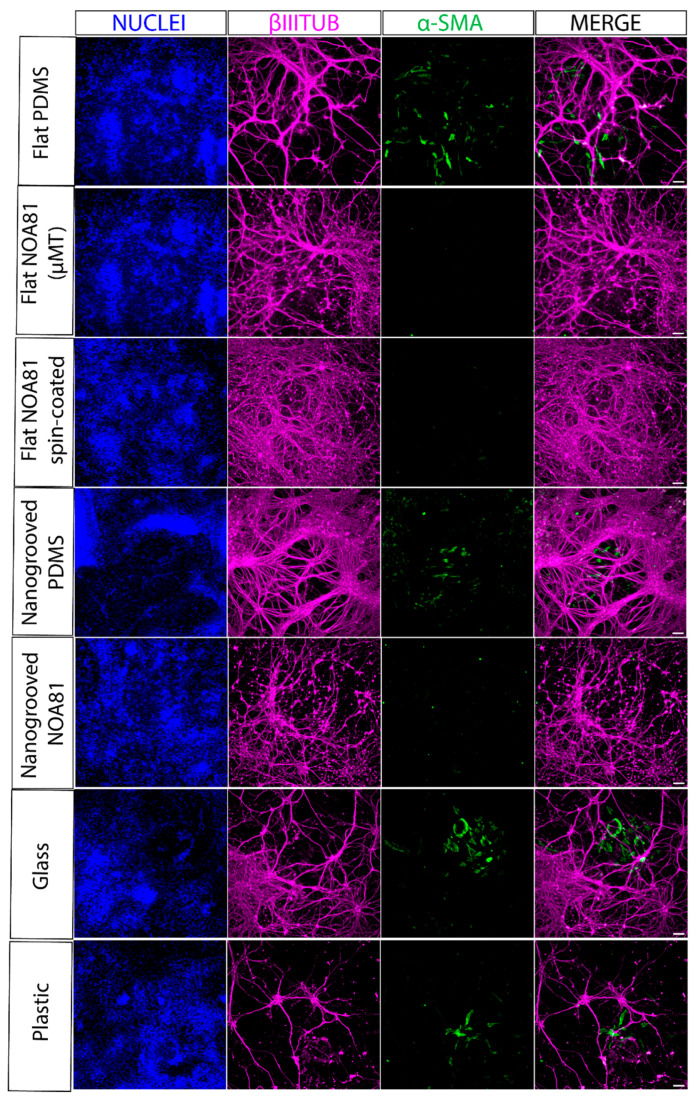
Immunofluorescence analysis of TUBB3 (enteric neuron marker) and α-SMA (myofibroblast marker) expression in day 21 ENS cultures generated following plating of day 9 spheres on the indicated substrates. Scale bars: 100 µm.

To investigate how surface topography and substrate material choice influence the differentiation of hiPSC toward enteric neurons and glia, we next compared differentiated ENS cultures grown on nanogrooved and flat substrates made from PDMS and NOA81, alongside flat glass and plastic substrates as controls. To enable sufficient cell material collection for gene expression analysis, we used modified coverslips made of PDMS and NOA81 with a larger culture area than standard MDs. To this end, we plated undissociated day 9 spheres (Figure 4a) in ENS-inducing culture conditions on modified coverslips incorporating flat or nanogrooved PDMS or NOA81 substrates fabricated by spin-coating or microtransfer molding (μTM). Following culture for a further 11 days, we assessed the induction of ENS progenitor/glial (*SOX10*, *S100β*) and enteric neuronal/subtype (*ASCL1*, *PRPH*, *PHOX2B*, *TH*, *CHAT*, *HTR2A*)-associated transcripts by reverse transcriptase quantitative real time qPCR (RT-qPCR); we found that the expression levels of these markers were comparable between all substrates used and similar to the controls (“glass”, “plastic”; Figure 5, Figure 6, Figure 7 and Figure 8). Interestingly, immunofluorescence analysis of the resulting cultures also revealed that use of the polymer NOA81 positively influences the elimination of contaminating α-SMA-positive myofibroblast-like cells. This effect may be attributed to changes in stiffness and ECM composition, which are well-established regulators of cell behavior and fate decisions, indicating that its employment could be an attractive strategy for improving the yield of hPSC-derived ENS cells.

These data confirm that WTC-11-derived cortical and ENS cells are biocompatible with the linear or radially arranged and compartmentalized microfluidic PDMS-based MDs, which are sealed against glass or other well-known nano- and microfabricated substrate materials, like PDMS or photosensitive NOA81. Together with the differentiated cells, these are the main design elements of the NoCs introduced in this paper. Furthermore, our results for ENS cells suggest that one can also seal the MD with other substrates with or without surface modification, as shown here with either flat or nanogrooved PDMS.

## 4. Conclusions

Our study successfully employed nano- and microfabrication techniques to develop MDs to guide and organize hiPSC-derived neural networks. This compartmentalized microfluidic platform supports both CNS and ENS cell cultures for NoC modeling. These MDs allow us to grow ENS/PNS cultures characterized by the expression of various neuronal and glial progenitor genes at the transcript level, as confirmed by PCR analysis of the ENS cells cultured on the different sealing substrates, such as NOA81 substrates. They potentially enable modulation of the cell type composition to favor specific ENS-derived cell types while reducing contamination by non-ENS cells, such as myofibroblasts, as demonstrated with the NOA81 substrates.

Our findings demonstrated that the neuronal networks within the MDs exhibited strong viability, expressed key representative markers, and formed synapses, while extending their axons into the microtunnels. The MDs also allow selective axonal outgrowth into wells that do not contain other cells, thereby making it a useful tool to study axonal outgrowth and morphology. Such a platform could be instrumental in the study of neurological diseases, offering a new avenue for understanding disease mechanisms and assessing therapeutic interventions. As demonstrated by larger area samples, utilizing different types of materials and topology in the microfabrication of MDs by means of microtransfer printing these to MD sealing substrates may further improve differentiation of hiPSC-derived enteric nervous system cell components, thanks to the reduction of myofibroblast contamination. These contaminating cells, marked by the expression of smooth muscle actin (α-SMA), represent an NC-derived alternative lineage and have been previously reported in similar NC-derived ENS cultures [45]. Their presence can be detrimental for the maintenance of mature enteric neurons over time, as they promote neuronal detachment from the well surface, ultimately leading to neuronal cell death. Therefore, minimizing such contamination is essential to sustain healthy and mature enteric neuronal networks in long-term culture.

In conclusion, the MD design aspects discussed in this paper can serve as a toolbox for investigating the pathology of neurodegenerative diseases like Parkinson’s Disease in more advanced Nervous System-on-Chip studies. Future work will focus on the co-culturing of different cell types within this platform to explore the potential for more complex neuronal network connectivity.

## Data Availability

All data supporting the findings of this study are available within the paper and Supplementary Materials or upon reasonable request from the corresponding author.

## Supplementary Materials

The following supporting information can be downloaded at: https://www.mdpi.com/article/10.3390/mi17020199/s1, Quantitative real-time PCR setup and primer details, fabrication of microtunnel device (MD) SU-8 mold, and incorporating cell culture reservoirs into MD using PMMA stencil. Figure S1: The layout of the photomask for photolithography (IDONUS, UV-EXP 150R tool, λ = 365 nm) to fabricate (a) radial (scale bar 1 mm) and (b) linear MDs including the tabulated tunnel width and pitch. The table further shows the details of different tunnels layout (scale bar 5 mm). (c,d) images show close-ups of (b) with a 10 μm tunnel width (scale bars 3 mm and 1 mm, respectively); Figure S2: The layout of the CAD used to fabricate the stencils for punching the MD PDMS parts to fabricate different MDs. (a) was used to prepare radial MDs with 2 mm reservoirs, (b) for fabricating radial MD with 3 mm reservoirs, and (c) was used for fabrication of 3 linear MDs (scale bar 6 mm. Dimensions in mm); Figure S3: Profilometer scan showing the height of the features on the SU-8 mold used in MD fabrication, indicating a tunnel height of approximately 4 µm, measured using a Dektak XT profilometer (Bruker Corporation, Billerica, MA, USA). Table S1: List of qPCR primer sets compatible with the Universal Probe Library system used for qPCR analysis.

## Abbreviations

The following abbreviations are used in this manuscript:

NoC	Nervous System-on-Chip
hiPSC	Human induced pluripotent stem cell
MD	Microtunnel device
CNS	Central nervous system
PNS	Peripheral nervous system
ENS	Enteric nervous system
NDD	Neurodegenerative disorder
NPC	Neural progenitor cell
NC	Neural crests
FACS	Fluorescence-activated cell sorting
RA	Retinoic acid
PBS	Phosphate-buffered saline
PFA	Paraformaldehyde
FCS	Fetal calf serum
BSA	Bovine serum albumin
PDMS	Polydimethylsiloxane
NOA81	Norland Optical Adhesive 81
COC	Cyclic olefin copolymer
μTM	Microtransfer molding
