# Supplementary material for: Nervous System-on-Chip: Innovative Microfluidic Platform to Compartmentalize hiPSC-Derived Neural Networks
Source: Micromachines (Basel). 2026 Feb 1;17(2):199. doi: 10.3390/mi17020199 (PMC12942585; doi:10.3390/mi17020199)
Supplement: Supplementary file 1 [file micromachines-17-00199-s001.zip › micromachines-4091788-supplementary.pdf]

Nervous System-on-Chip: Innovative Microfluidic Platform to Compartmentalize hiPSC-Derived Neural Networks

Rahman Sabahi-Kaviani, Antigoni Gogolou, Celine Souilhol, Mark van der Kroeg, Steven A. Kushner, Femke M. S. de Vrij, Anestis Tsakiridis and Regina Luttge

S1. Quantitative real-time PCR setup and primer details

Table S1. List of qPCR primer sets compatible with the Universal Probe Library system used for qPCR analysis.

Gene	Forward primer	Reverse primer	Roche UPL Probe
GAPDH	5'-agccacatcgctcagacac-3'	5'-gcccaatacgaccaaatcc-3'	60
SOX10	5'-ggctcccccatgtcagat-3'	5'-ctgtcttcggggtggttg-3'	21
S100β	5'-gagctttcccatttcttagagga-3'	5'- gaagtcacattcgccgtctc3'	47
ASCL1	5'-cgacttcaccaactggttctg-3'	5'-atgcaggttgtgcgatca-3'	38
PHOX2B	5'ctaccccgacatctacactcg-3'	5'-ctcctgcttgcgaaacttg-3'	17
PRPH	5'-aagacgactgtgcctgaggt-3'	5'- tgctccttctgggactctgt-3'	10
TRKC	5'- ccgtacgagagggtgacaat-3'	5'- tggtccagttcagattggtct-3'	21
TH	5'-acgccaaggacaagctca-3'	5'-agcgtgtacgggtcgaact-3'	42
HTR2A	5'- tgatgtcacttgccatagctg-3'	5'- caggtaaatccagactgcacaa-3'	3
CHAT	5'-cagccctgatgccttcat-3'	5'-cagtcttcgatggagcctgt-3'	78

S2. Fabrication of microtunnel device (MD) SU-8 mold

The details of the photolithography procedure utilized for the fabrication of microtunnel devices (MDs) mold using SU-8 2010 (Microchem Inc., Newton, MA, United States) on a 4-inch Si wafer is as following. After spin-coating the resist onto a silicon wafer (type, supplier) it was soft-baked at 95 °C for 140 s on a hot plate (EchoTherm™, HS60 series) and then exposed to collimated UV light (IDONUS, UV-EXP 150R, Neuchatel, Switzerland) through a foil photomask (designed in AutoCAD 2022, printed by CAD/Art Services Inc., Bandon, OR, United States) for 220 mJ/cm^2. The mask layout is depicted in Fig. S1. Post-exposure bake took place at 95 °C for 3.5 min again on the same hot plate. Subsequently, the latent image in the resist was developed in a beaker containing developer (mrDev-600, Microresist Technology GmbH), washed and dried. The SU-8 structures thickness was measured using a profilometer (Bruker Dektak XT, Massachusetts, USA). The mold was put into a petri dish (Square petri dish, 120mm, vented, Greiner Bio-One International GmbH, the Netherlands) and kept at room temperature in a lab cabinet to shield it from any external contaminants such as dust prior to its further use.

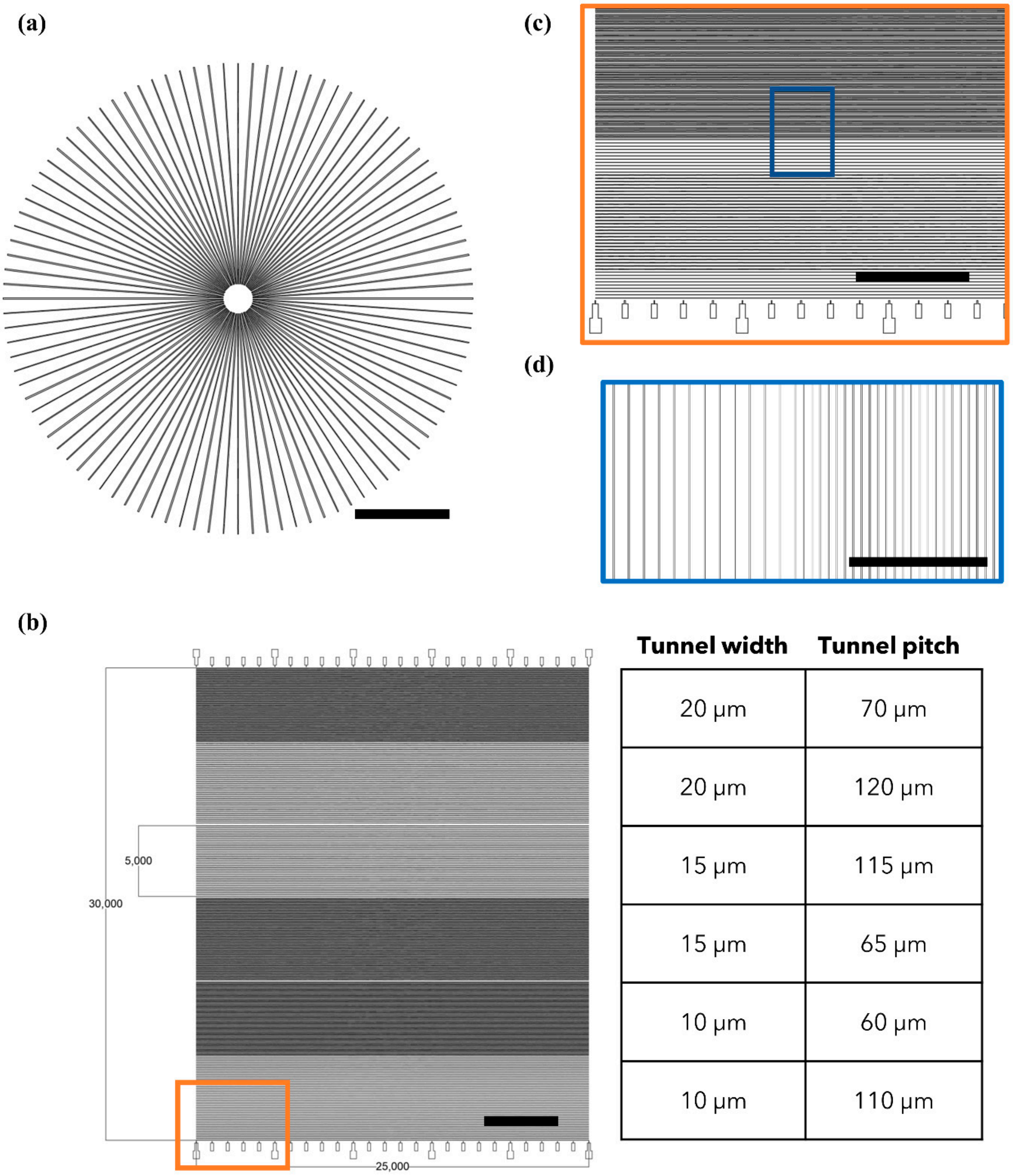

Tunnel width	Tunnel pitch
20 µm	70 µm
20 µm	120 µm
15 µm	115 µm
15 µm	65 µm
10 µm	60 µm
10 µm	110 µm

Figure S1. The layout of the photomask for photolithography (IDONUS, UV-EXP 150R tool, λ = 365 nm) to fabricate (a) radial (scale bar 1 mm) and (b) linear MDs including the tabulated tunnel width and pitch. The table further shows the details of different tunnels layout (scale bar 5 mm). (c,d) images show close-ups of (b) with a 10 µm tunnel width (scale bars 3 mm and 1 mm, respectively).

S3. Incorporating cell culture reservoirs into MD using PMMA stencil

After peeling off the PDMS from the mold, cell culture reservoirs were punched using a biopsy tool from the side of the PDMS that exposes the microtunnels assisted by applying a PMMA stencil. The stencil was first aligned with PDMS MD under stereomicroscope assuring that the central circle in the PMMA stencil (Figs. S2a-b) is located exactly in the center of the radial MD (Fig. S1a). Consequently, the stencils was placed on top of MD and was secured with tapes so that it would not move during punching process. Finally, a 2-mm or 3-mm biopsy punch was used to create the desired reservoirs in the designated locations on MD. This PMMA stencil with pre-set locations of the reservoirs was made by

laser cutting (Universal Laser System, VLS Model 3.50, ENGRAVING SYSTEMS, LLC, Connecticut, USA) from a 1-mm polymethyl- methacrylate (PMMA) sheet (Precision acrylic glass, transparent, colourless, Modulor, Germany). Before using the PMMA as a stencil, the laminated protective foil was removed. The stencil was then washed in DI water and dried to remove any debris that might have resulted from the laser cutting process on the PMMA. The radial MDs feature one reservoir in the center and five additional reservoirs situated at a distance of 2.3 mm from the central reservoir equally angularly spaced along the circumference. The distance between the reservoirs in the linear MD is 4 mm. The Computer Aided Design (CAD) file for the stencil used in this research to incorporate the necessary reservoirs at the designated positions in both radial and linear MDs is given in Fig. S2.

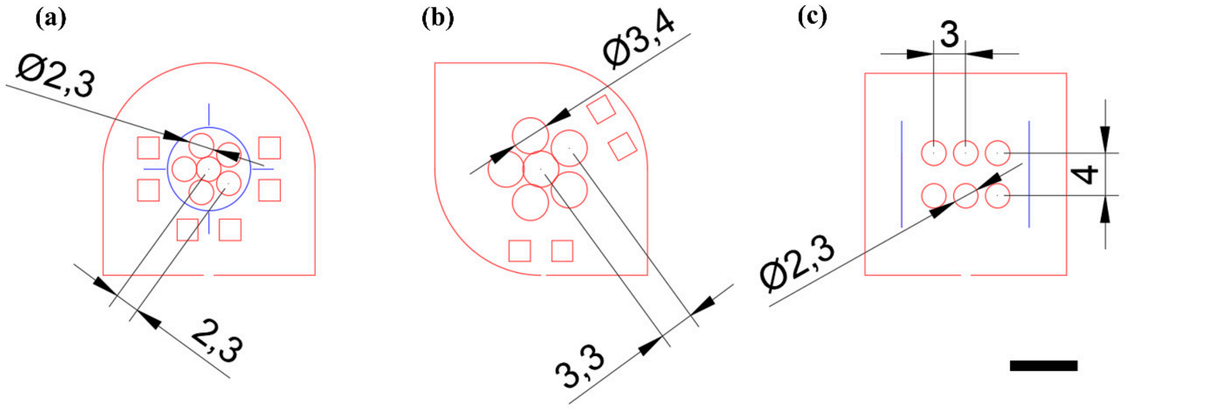

Figure S2. The layout of the CAD used to fabricate the stencils for punching the MD PDMS parts to fabricate different MDs. (a) was used to prepare radial MDs with 2 mm reservoirs, (b) for fabricating radial MD with 3 mm reservoirs, and (c) was used for fabrication of 3 linear MDs (scale bar 6 mm. Dimensions in mm).

S4. Profilometer characterization of SU-8 mold

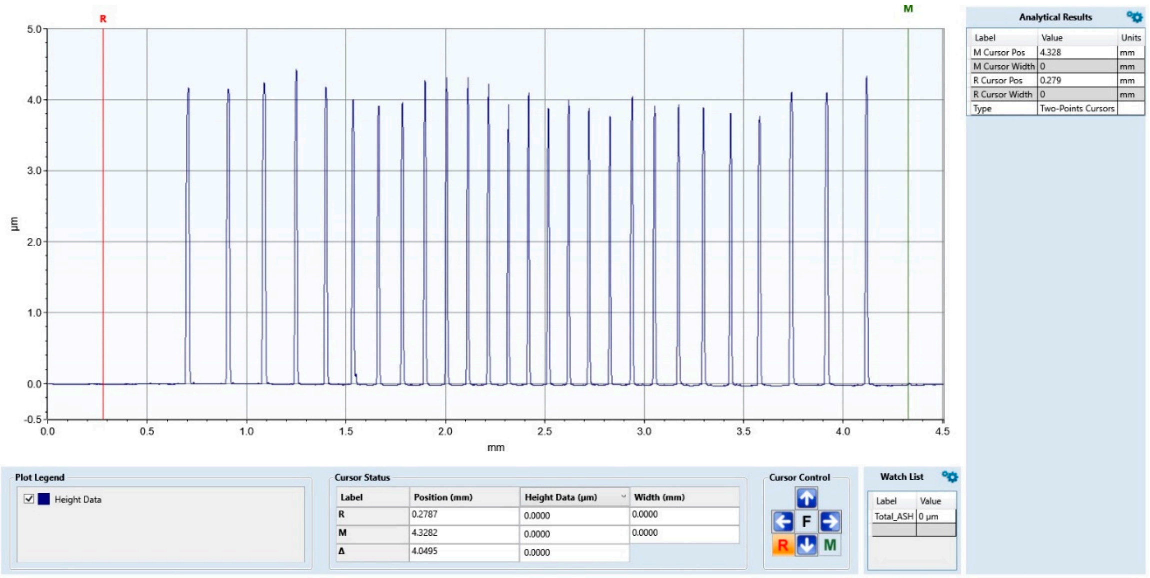

Figure S3. Profilometer scan showing the height of the features on the SU-8 mold used in MD fabrication, indicating a tunnel height of approximately 4 µm, measured using a Dektak XT profilometer (Bruker Corporation, Billerica, MA, USA).
